# The Physiological Functionality of PGR5/PGRL1-Dependent Cyclic Electron Transport in Sustaining Photosynthesis

**DOI:** 10.3389/fpls.2021.702196

**Published:** 2021-07-07

**Authors:** Mingzhu Ma, Yifei Liu, Chunming Bai, Yunhong Yang, Zhiyu Sun, Xinyue Liu, Siwei Zhang, Xiaori Han, Jean Wan Hong Yong

**Affiliations:** ^1^College of Land and Environment, National Key Engineering Laboratory for Efficient Utilization of Soil and Fertilizer Resources, Northeast China Plant Nutrition and Fertilization Scientific Observation and Research Center for Ministry of Agriculture and Rural Affairs, Key Laboratory of Protected Horticulture of Education Ministry and Liaoning Province, Shenyang Agricultural University, Shenyang, China; ^2^The UWA Institute of Agriculture, The University of Western Australia, Perth, WA, Australia; ^3^School of Biological Sciences, The University of Western Australia, Perth, WA, Australia; ^4^School of Agriculture and Environment, The University of Western Australia, Perth, WA, Australia; ^5^National Sorghum Improvement Center, Liaoning Academy of Agricultural Sciences, Shenyang, China; ^6^Professional Technology Innovation Center of Magnesium Nutrition, Yingkou Magnesite Chemical Ind Group Co., Ltd., Yingkou, China; ^7^Department of Biosystems and Technology, Swedish University of Agricultural Sciences, Alnarp, Sweden

**Keywords:** photosynthesis, cyclic electron transport, proton gradient regulation 5, PRG5-like photosynthetic phenotype 1, photoinhibition

## Abstract

The cyclic electron transport (CET), after the linear electron transport (LET), is another important electron transport pathway during the light reactions of photosynthesis. The proton gradient regulation 5 (PGR5)/PRG5-like photosynthetic phenotype 1 (PGRL1) and the NADH dehydrogenase-like complex pathways are linked to the CET. Recently, the regulation of CET around photosystem I (PSI) has been recognized as crucial for photosynthesis and plant growth. Here, we summarized the main biochemical processes of the PGR5/PGRL1-dependent CET pathway and its physiological significance in protecting the photosystem II and PSI, ATP/NADPH ratio maintenance, and regulating the transitions between LET and CET in order to optimize photosynthesis when encountering unfavorable conditions. A better understanding of the PGR5/PGRL1-mediated CET during photosynthesis might provide novel strategies for improving crop yield in a world facing more extreme weather events with multiple stresses affecting the plants.

## Introduction

Life on earth depends on energy derived from the sun. Photosynthesis is the pivotal process that could harvest light energy and ultimately generate biomass using water, CO_2_ and mineral nutrients. The bulk of our earth’s energy resources is derived from global photosynthetic activity in either recent or ancient times (Vass et al., 2007; Liu et al., 2011; Lambers and Oliveira, 2019; Nawrocki et al., 2019). The normal operation of photosynthesis is inseparable from the participation of light energy, but any excessive high light would impact the photosystem II (PSII), photosystem I (PSI) and the other thylakoid membrane proteins and resulting in photoinhibition and the accumulation of reactive oxygen species (ROS; Foyer and Noctor, 1999; Chaux et al., 2017; Liu, 2020). In the real world, multiple stress episodes affecting growth and development are common (Suzuki et al., 2014). Any excessive high light situation could be exacerbated further with a co-occurring high temperature (Sun et al., 2017; Lu et al., 2020), low temperature (Liu et al., 2013; Song et al., 2020; Wu et al., 2020), phosphorus deficiency (Carstensen et al., 2018; Shi et al., 2019) and drought (Wada et al., 2019). Plants have evolved several adaptations to cope with the unfavorable light situation: adjusting leaf orientation, ROS scavenging competence (Gill and Tuteja, 2010), xanthophyll cycle (Kuczynska et al., 2020), state transitions strategy (Hepworth et al., 2021), cyclic electron transport (CET; Yadav et al., 2020) and photorespiration (Storti et al., 2019). This review summarizes the main biochemical processes of PGR5/PGRL1-dependent CET pathway. The significance of the PGR5/PGRL1-dependent CET pathway is discussed to understand how plants optimize photosynthesis under unfavorable conditions by protecting the PSII and PSI, ATP/NADPH ratio maintenance, and regulating the transitions between linear electron transport (LET) and CET.

## Photosynthetic Electron Transport

**Figure 1:**
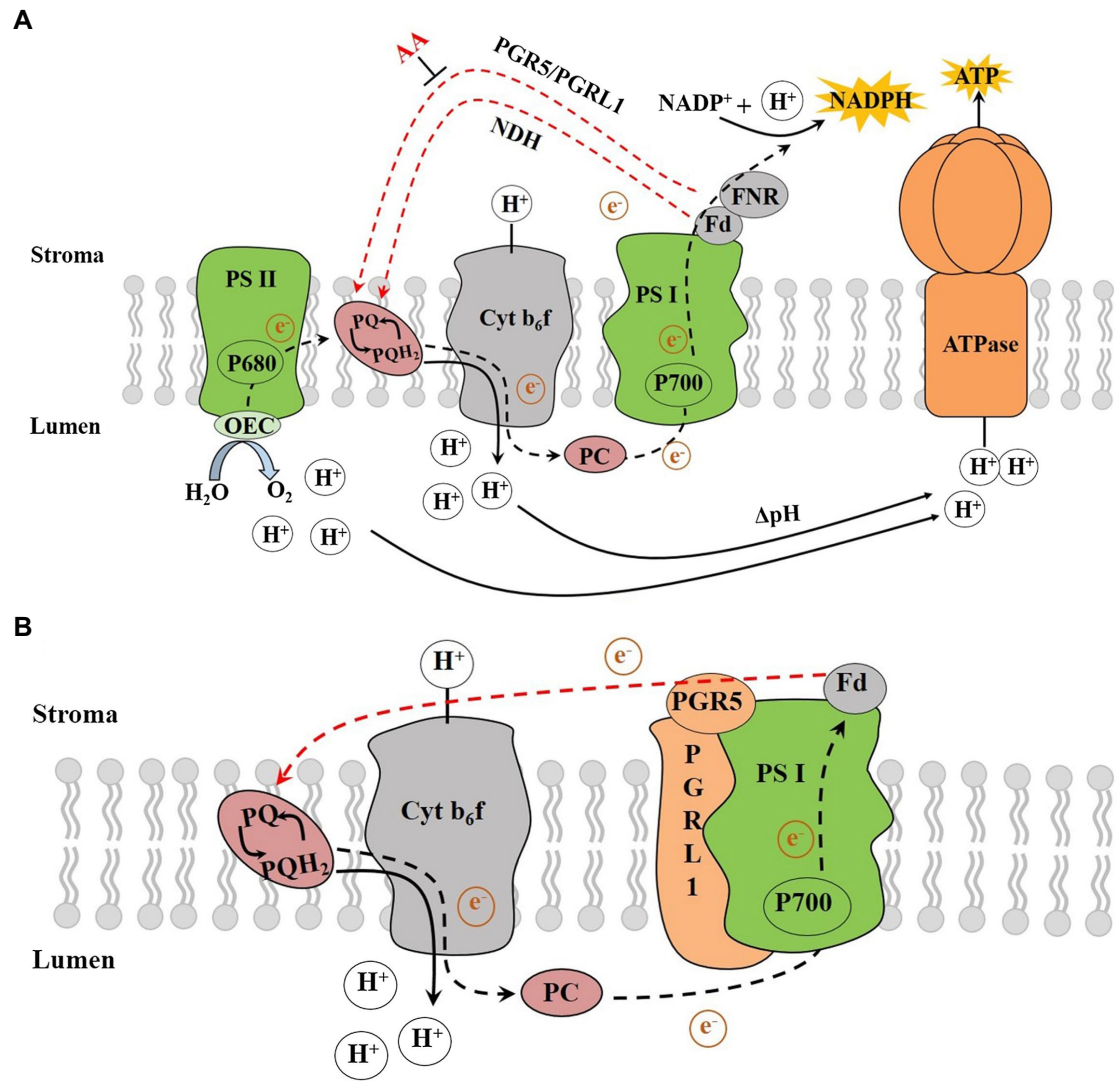
**(A)** The photosynthetic electron transport chain and **(B)** The PGR5/PGRL1-dependent CET. AA, antimycin A; ATPase, ATP synthase; Cytb_6_f, cytochrome b_6_f complex; Fd, ferredoxin; FNR, ferredoxin-NADP reductase; Lumen, thylakoid lumen; NADPH, reduced nicotinamide adenine dinucleotide phosphate; NDH, NADH dehydrogenase-like; OEC, oxygen-evolving complex; P680, pigment molecule of PSII reaction centre; P700, pigment molecule of PSI reaction centre; PC, plastocyanin; PGR5, proton gradient regulation 5; PGRL1, PRG5-like photosynthetic phenotype 1; PQ, plastoquinone; PQH_2_, plastoquinol; PSI, photosystem I; PSII, photosystem II, Stroma, thylakoid stroma and ΔpH, proton gradient (adapted from Yamori and Shikanai, 2016).

Chloroplasts convert light energy into chemical energy *via* electron transport (ET), which provides energy for the Calvin cycle and other processes. During the LET, electrons derived from water splitting in PSII are transferred *via* the cytochrome (Cytb_6_f) complex, PSI and ferredoxin (Fd) to the ferredoxin-NADP reductase (FNR), which ultimately reduce NADP^+^ to NADPH, resulting in the production of NADPH (Figure 1A; Lu et al., 2020). The H^+^ enters the thylakoid lumen by the Q cycle, and the H^+^ produced by water splitting in OEC together to form the required proton motive force (*pmf*) across the thylakoid membrane (Wang et al., 2020). The *pmf*, composed of the transmembrane potential (Δ*ψ*) and proton gradient (ΔpH), plays a key role in driving the chloroplast ATP synthase to synthesize ATP (Lapashina and Feniouk, 2018). ATP synthesis coupled with the LET is known as noncyclic photophosphorylation (NCPSP; Arnon et al., 1954, 1958). The energy derived from LET and NCPSP plays an essential role in photosynthesis and other processes. However, there would be insufficient ATP from the LET during certain multiple stress situations. Plants could compensate for the deficiency of ATP/NADPH in the LET by using the CET around the PSI (Sato et al., 2019; Ma et al., 2021), the water-water cycle (Mehler reaction; Asada et al., 1999) and the mitochondrial alternative oxidase respiration (Meng et al., 2012).

## Cyclic Electron Transport

The pathway of electron transport around PSI, which recycles electrons from Fd to PQ, is called the CET, while the ATP synthesis coupled with it is called the cyclic photophosphorylation (Bendall and Manasse, 1995). Moss and Bendall (1984) proposed that an antimycin A (AA)-sensitive enzyme is involved in the ET from Fd to PQ termed as the ferredoxin-plastoquinone reductase (FQR) with the following configuration: PSI-Fd-FQR-PQ-Cyt b_6_f-PSI. The discovery of a protein complex that could receive electrons from Fd and transferring electrons to PQ represented major progress in CET research. Due to the high similarity to complex I within the mitochondrial respiratory chain, it was aptly called NDH (NADH dehydrogenase-like complex; Burrows et al., 1998). The NDH pathway is the main pathway compared with PGR5 one in cyanobacteria (Miller et al., 2021). It has been reported that NDH pathway plays a crucial role at high temperature or low temperature in tobacco (Wang et al., 2006) and low-light intensity in rice (Yamori et al., 2015) and *Marchantia polymorpha* (Ueda et al., 2012). However, it is not sensitive to AA, which implied that there is possibly another FQR pathway that is sensitive to AA and regulated by Fd in CET.

### The PGR5/PGRL1-Mediated CET Pathway

It was suggested that the CET around PSI helps contribute electrons to synthesize ATP: *Chlamydomonas* (Yadav et al., 2020), *Phaeodactylum* (Zhou et al., 2020), C_3_ (Wang et al., 2015) and C_4_ plants (Munekage et al., 2010). There are at least two CET pathways in vascular plants and *Phycophyta*: antimycin A-sensitive pathway that involves proton gradient regulation 5 (PGR5) and PGR5-like photosynthetic phenotype 1 (PGRL1), and antimycin A-insensitive NADH dehydrogenase-like (NDH) pathway (Figure 1A; Munekage et al., 2002; Huang et al., 2005; Dalcorso et al., 2008; Taira et al., 2013; Ishikawa et al., 2016). There is a high similarity between the NDH and respiratory chain proteins. Conversely, PGR5 has no homology with the mitochondrial respiratory chain proteins. In a mutant of *pgr5*, due to less influx of protons that should be from the Q cycle, the ability of non-photochemical quenching (NPQ) PSII is reduced under strong light (Yadav et al., 2020). Although PGR5 plays a key role in CET from Fd to PQ, its molecular characteristics are not sufficient to deliver all the functionality reported for FQR. Specifically, PGR5 does not contain any redox-active cysteine residues that mediates ET nor has any transmembrane domains (Yamori and Shikanai, 2016). Therefore, the role played by PGR5 in the AA-sensitive CET pathway is still unclear. It was suggested that the decrease of CET activity in *pgr5* mutants is due to its plausible role in feedback regulation (Nandha et al., 2007), and the postulated function of PGR5 is to regulate LET (Suorsa et al., 2012).

The PGRL1 was identified as another important regulator of the CET process in *Arabidopsis*. Plants lacking PGRL1 showed a decrease in CET rate and exhibiting a similar performance to the *pgr5* mutant (Dalcorso et al., 2008; Yadav et al., 2020). The regulatory role of PGR5/PGRL1-dependent CET under environmental perturbations has been studied (Wang et al., 2014; Yamori et al., 2016; Wolf et al., 2020). PGR5 is a small thylakoid protein without any known motifs that suggest its function (Munekage et al., 2002), while PGRL1 is a transmembrane protein with two transmembrane domains, and its two cysteine residues are involved in an iron cofactor binding (Hertle et al., 2013). The previous studies showed that the PGR5 proteins in *Arabidopsis* have low similarity to those found in cyanobacteria, excluding the coding genes of PGRL1 (Peltier et al., 2010). The double mutant of *Arabidopsis prgl1ab* showed a phenotype similar to that of *pgr*5 (Dalcorso et al., 2008). In rice *pgr*5 mutants, the PGRL1 protein level decreased by 50% (Nishikawa et al., 2012). Generally, the transport of electrons from Fd to PGRL1 requires the participation of PGR5 proteins, where the loss of any protein would affect the CET activity (Munekage et al., 2002; Dalcorso et al., 2008; Kono et al., 2014). Under *in vitro* conditions and when reduced Fd is present, an unknown redox reaction would catalyse the formation of disulphide bonds between cysteine residues in PGRL1 and the recombinant PGRL1 thereby reducing the analogue of PQ and quinone 2,6-dimethyl-p-benzoquinone (Hertle et al., 2013). This finding was confirmed by examining the mercaptan group of FQR (Strand et al., 2016). Shikanai (2007) speculated that PGR5 and PGRL1 proteins are important components of FQR. Subsequently, when the molecular features of PGRL1 were found to be similar to the FQR protein, the researchers further proposed that PGRL1 could be the FQR proteins (Figure 1B; Hertle et al., 2013; Labs et al., 2016).

### State Transitions and the CET

In plants, the redistribution of excitation energy between the two photosystems is modulated by reversible phosphorylation of light-harvesting complex II (LHCII) in response to light fluctuation (Allen et al., 1981; Bhatti et al., 2020). Generally, these processes are known as state transitions (Bonaventura and Myers, 1969). For *Chlamydomonas reinhardtii*, when PSII is excited, the LHCII is phosphorylated, separated from PSII and adhering to the PSI. Meanwhile, the absorbed light energy is allocated to PSI, and thereby allowing the CET to dominate; this is called state II. PSI is preferentially excited during state I during which LHCII-P is dephosphorylated, recombined with PSII, and giving priority to facilitate the LET (Finazzi et al., 2002). While it is true that CET is the main pathway of ET during state II, this does not imply that state II is a necessary condition for the CET to operate. It was found in *C. reinhardtii* and *Arabidopsis* that the CET is not affected by the state transition (Takahashi et al., 2013). Although the CET is not related to state transition, state II is beneficial for the separation of the CET-PSI complexes (Yamori and Shikanai, 2016). The core mechanism of PGR5/PGRL1-mediated CET is similar in *C. reinhardtii* and *Arabidopsis* (Yamori and Shikanai, 2016), except for their supercomplex components related to ET. Iwai et al. (2010) identified the supercomplexes containing FNR, Fd, PGRL1, Cytb_6_ and PSI in *C. reinhardtii*. In *Arabidopsis*, however, it was only confirmed that the PGRL1-PGR5 complex could interact with PSI, thus facilitating the formation of *Arabidopsis* CET supercomplex (Dalcorso et al., 2008). Although the potential CET supercomplex was not identified clearly, there were more studies to support the association of PGR5 and PGRL1 in the CET (Breyton et al., 2006; Xue et al., 2017).

## The Function of PGR5/PGRL1-Mediated CET in Plants

### Regulating the Level of ATP and Maintaining the Balance of ATP/NADPH

The ‘Proton Gradient Regulation 5’ or PGR5 plays a pivotal role in proton gradient regulation. In the chloroplasts, the regulation of *pmf* must satisfy two competing physiological demands: (1) ensuring the requirements of carbon fixation for ATP and (2) decreasing the ET rate to avoid light damage under certain situations. Under relatively low light, the proportion of Δ*ψ* is equal to that of ΔpH. With increasing light, the proportion of ΔpH in *pmf* increases gradually. In *Arabidopsis pgr5* mutants, ΔpH accounts for about 90% of the total *pmf* at a light intensity greater than 312 μmol·m^−2^ s^−1^ (Yamamoto et al., 2016). Therefore, the size of ΔpH may be partly compensated by increasing the partitioning of ΔpH in *pmf* in *pgr5* (Yamamoto et al., 2016). To move from Δ*ψ* to ΔpH, some cations (mainly Mg^2+^ and K^+^) have to be transported to the stroma *via* the thylakoid membrane (Kramer et al., 2003). Hind et al. (1974) indicated that the outflow of these cations could facilitate *pmf* adjusting into the ΔpH form. Besides, AtVCCN1, a voltage-dependent chloride channel, located in *Arabidopsis* thylakoid membrane can make Cl^−^ influx into the lumen during illumination and partially dissipate the Δ*ψ* in the lumen, thereby increasing the ΔpH/Δ*ψ* ratio (Herdean et al., 2016).

**Figure 2:**
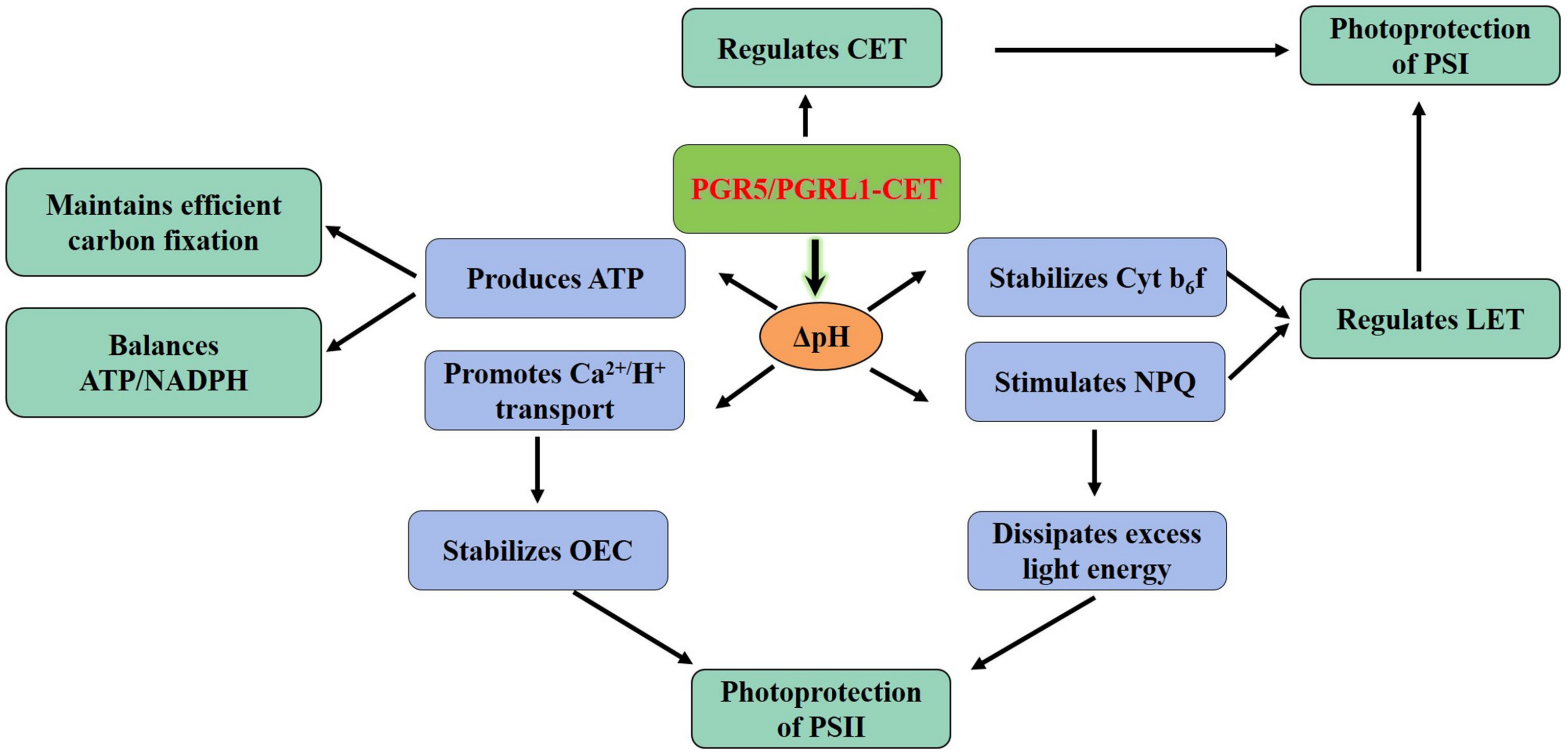
Physiological functionality of the PGR5/PGRL1-dependent CET. The PGR5/PGRL1-mediated CET produces more ATP by increasing ΔpH to balance ATP/NADPH and meeting the requirements for efficient C fixation. Meanwhile, the increase of ΔpH would protect the PSII from photoinhibition *via* stimulating the NPQ and facilitating the transport of Ca^2+^/H^+^. ATP, adenosine triphosphate; CET, cyclic electron transport; Cytb_6_f, cytochrome b_6_f complex; LET, linear electron transport; NADPH, reduced nicotinamide adenine dinucleotide phosphate; NPQ, non-photochemical quenching; OEC, oxygen-evolving complex; PGR5, proton gradient regulation 5; PGRL1, PRG5-like photosynthetic phenotype 1; PSI, photosystem I and PSII, photosystem II.

Plants regulate the proportion of ATP/NADPH to meet the competing demands of metabolism and photoprotection. Thus, the regulation of the electron distribution between LET and CET is essential to maintaining optimal photosynthesis under prevailing conditions. Particularly, the PGR5/PGRL1-dependent CET plays a central role in the regulation of LET *via* the downregulation of the Cyt b_6_f complex (Shikanai, 2014; Yamori et al., 2016). The function of CET is more relevant under conditions when the LET cannot produce sufficient ΔpH; consequently, it is necessary to improve the ratio of ATP/NADPH by increasing the ΔpH to promote the synthesis for more ATP (Figure 2; Yamori and Shikanai, 2016). Besides, sufficient ΔpH of thylakoid lumen contributes to the downregulation of electron transport through NPQ, preventing photodamage (Niyogi, 1999; Shikanai, 2007). Both PGR5/PGRL1 and NDH-mediated CET play a role in low light and facilitating CO_2_ assimilation by providing additional ATP (Nishikawa et al., 2012). In contrast, the regulatory effect of PGR5/PGRL1 and NDH-mediated CET on ATP/NADPH is negligible in rice-growing under strong light (Yamori et al., 2015). However, strong light led to the decrease of *pmf* formation in *pgr*5 mutant in *Arabidopsis* and the concomitant decrease of ATP yield, thereby disrupting the optimal ATP/NADPH balance (Kawashima et al., 2017). The stimulation of ATP/NADPH homeostasis in primary metabolism demonstrated that the energy requirement under high light is not less than that under low light (Walker et al., 2014). Hence, one might envisage that the CET should be beneficial to regulating the balance of ATP/NADPH under different light conditions. Recent studies have shown that the CET was needed to achieve a balanced ATP/NADPH ratio even under non-stress conditions in C_3_ plants (Wang et al., 2015).

### Inducing the NPQ and Protecting the PSII

Any excess light during photosynthesis would lead to photo-oxidative damage and reducing carbon fixation (Paredes and Quiles, 2017). The non-photochemical quenching mechanism (NPQ) plays an essential role in the photoprotection mechanism (Erickson et al., 2015). Inducing the qE component of NPQ to dissipate excessive absorbed light energy is dependent on the thylakoid lumen acidification modulated by the CET (Müller et al., 2001; Johnson et al., 2014). Under multiple stresses, electrons will preferentially reduce Fd and NADP^+^ and not O_2_, thereby avoiding oxidative damage to the photosystems caused by the excess light energy (Chow and Hope, 2004; Kukuczka et al., 2014). The previous studies have shown that NDH-mediated CET plays a significant role in rice and *M. polymorpha* under low light (Ueda et al., 2012; Yamori et al., 2015). However, in *Arabidopsis*, the deletion of the NDH gene did not alter photosynthesis significantly (Hashimoto et al., 2003). It was only when both PGR5 and NDH were mutated that the seedling has an altered phenotype (Munekage et al., 2004). Therefore, for C_3_ plants, the PGR5/PGRL1 pathway is the major pathway of CET (Munekage et al., 2004; Okegawa et al., 2008; Wang et al., 2015; Okegawa and Motohashi, 2020). Recent studies have indicated that the role of CET probably varies with light intensity (Huang et al., 2015). The generation of CET-dependent *pmf* is for the synthesis of ATP under lower light (Avenson et al., 2005; Walker et al., 2014). With higher light, the acidification of thylakoid lumen is beneficial to protect PSI and PSII from photoinhibition (Takahashi et al., 2009; Tikkanen et al., 2014).

Both *pgr*5 and *pgrl*1 mutants in *Arabidopsis* were sensitive to abiotic stress, such as high light and extreme temperature (Munekage et al., 2002; Jin et al., 2017; Kawashima et al., 2017). When the PSII repair function of wild-type and *pgr*5 mutants was inhibited, compared with wild-type plants, PSII in *pgr*5 mutants was still more sensitive to strong light (Takahashi et al., 2009), indicating that PGR5 deficiency caused photodamage to photosystems (Okegawa et al., 2010). The PSI is the likely primary target of photoinhibition, and the dynamic balance between photodamage and restoration in PSII maintains its stability (Pospsil and Tyystjarvi, 1999). Generally, the protection of PSII by CET mediated by PGR5/PGRL1 under adverse environments involves at least two different mechanisms. Firstly, the acidification of the thylakoid lumen activates NPQ to dissipate excess light energy, thereby reducing ROS production in the PSII complex (Munekage et al., 2008). Secondly, the formation of ΔpH promotes the reversed Ca^2+^/H^+^ transport to increase the concentration of Ca^2+^ in the thylakoid lumen (Ettinger et al., 1999). As the stability of OEC depended on the level of lumen Ca^2+^ (Krieger and Weis, 1993), the acidification in the lumen would avoid the photodamage of PSII by increasing the stability of OEC (Figure 2; Takahashi et al., 2009; Huang et al., 2016). Notably, the effects of PGR5 overexpression were strikingly pleiotropic. The accumulation of PGR5 could enhance the high-light resistance of the plants, but it also markedly delayed the greening of cotyledons, thereby causing the slower seedling growth in the initial growth stage (Okegawa et al., 2007; Long et al., 2008; Sugimoto et al., 2013).

### Regulating the LET and Protecting the PSI

The Fe-S clusters within the PSI complex are vulnerable to ROS when exposed to fluctuating light. In particular, PSI photodamage occurred before PSII in *pgr*5 mutants (Sonoike, 2011; Suorsa et al., 2012; Kono et al., 2014, 2017). Unlike the effective and fast repair of PSII, the restoration of PSI is slower and consequently. In general, most PSI damages are considered to be almost irreversible (Zivcak et al., 2014). Gollan et al. (2017) found that PSI damage inhibited carbon fixation and other processes after high-light exposure.

Similar to its role in PSII, the protective effect of PGR5/PGRL1-mediated CET on PSI is related to the formation of ΔpH (Yamamoto and Shikanai, 2019). The CET-dependent ΔpH formation not only contributes to the synthesis of ATP but also regulates the ET *via* acidifying the thylakoid lumen (Shikanai, 2014, 2016; Yamori and Shikanai, 2016). The PSI acceptor-side regulation by CET sustains electron sinks downstream of PSI and preventing the over-reduction of the PSI (Munekage et al., 2002). The acidification of the thylakoid lumen downregulates the Cyt b_6_f complex thereby slowing down the ET from PSII towards PSI and induces the thermal dissipation of absorbed excess photon energy from the PSII antennae (Shikanai, 2016; Yamamoto and Shikanai, 2019). This is the PSI donor-side regulation by CET for PSI photoprotection (Suorsa et al., 2012). It has been reported that exogenous calcium alleviates nocturnal chilling-induced photo damage by facilitating CET, thereby enhancing the photosynthesis and biomass accumulation of peanut under low nocturnal temperature stress (Song et al., 2020; Wu et al., 2020). Additionally, plant dry weight was significantly lowered in the rice *PGR*5-knockdown line compared to that of WT, especially under fluctuating light (Yamori et al., 2016). Therefore, stimulating CET *via* artificial growth regulation might be a novel strategy to maintain sufficient photosynthetic carbon fixation and enhance yield under unfavorable conditions. Most notably, Rantala et al. (2020) indicated that both PGR5 and NDH-1 systems do not function as protective electron acceptors but mitigate the consequences of PSI inhibition and protected the remaining PSI centres by enhancing pH-dependent regulation of electron transfer from PSII to PSI.

Functional analysis showed the PSI remained fully reduced under high light in *pgr*5 mutants. Interestingly, in the wild type under high light, the PSI complex was oxidized; the damage of PSI in *pgr*5 was later mitigated by exogenous application of 3-(3,4-dichlorophenyl)-1,1-dimethylurea (DCMU: inhibitor of the PSII to PSI ET; Tikkanen et al., 2010). These observations implied that the PGR5/PGRL1-mediated CET could reduce PSI damage from excessive electron flow under strong light. PGR5 and PGRL1 play crucial roles in the efficient operation of CET, whereas the maximum rate of CET is only slightly affected in *pgr*5 mutants (Nandha et al., 2007). Although the CET varies slightly, the change of ATP/NADPH ratio would be sufficient to have a substantial impact on the levels of ADP, phosphatidylinositol (Pi) and NADP^+^, thus reducing the activity of PSI electron acceptor and modulating the rate of LET (Kramer et al., 2004; Avenson et al., 2005; Suorsa et al., 2016). This evidence highlighted the important role of PGR5 in regulating the LET to CET transition (Figure 2; Suorsa et al., 2016).

## Future Outlook

Optimizing photosynthesis is an effective way to improve plant productivity. However, the variation of light and environmental conditions would often lower photosynthetic capacity and hampering electron transmission. The PGR5/PGRL1-dependent CET around PSI plays an important homeostatic role in electron transfers and thereby alleviating photoinhibition. With the recent advent of molecular techniques and sensitive analytical tools, scientists have achieved a better understanding of the PGR5/PGRL1-CET putative structure and functionality. Thus, the biological significance of the PGR5/PGRL1 pathway is better understood now although there exist several unsolved questions: pathway initiation and interactions leading to better efficiency; the relationship between PGR5/PGRL1 and FQR and the effect of PGR5/PGRL1 expression on the PSII. Most published studies have focused on *Arabidopsis* and rice, and with less emphasis on other crops. With the availability of novel research tools, it is possible to elucidate the complex regulatory network of the PGR5/PGRL1 pathway and its role in optimizing photosynthesis under unfavorable conditions.

## Author Contributions

YL, MM, XH, and JY are responsible for the general overview of the opinions stated in the manuscript. YL, MM, CB, YY, ZS, XL, SZ, and JY wrote and modified the manuscript. All authors reviewed and approved the final version of the submitted manuscript.

### Conflict of Interest

YY was employed by the company Yingkou Magnesite Chemical Ind Group Co. Ltd.

The remaining authors declare that the research was conducted in the absence of any commercial or financial relationships that could be construed as a potential conflict of interest.
